# Clinical Trials Based on Mesenchymal Stromal Cells are Exponentially Increasing: Where are We in Recent Years?

**DOI:** 10.1007/s12015-021-10231-w

**Published:** 2021-08-16

**Authors:** Umberto Galderisi, Gianfranco Peluso, Giovanni Di Bernardo

**Affiliations:** 1 Department of Experimental Medicine, Luigi Vanvitelli Campania University, Naples, Italy; 2 Center for Biotechnology, Sbarro Institute for Cancer Research and Molecular Medicine, Temple University, Philadelphia, PA USA; 3 Genome and Stem Cell Center (GENKÖK), Erciyes University, Kayseri, Turkey; 4 Research Institute On Ecosystems (IRET), CNR, Naples, Italy

**Keywords:** Mesenchymal stem cells, Stem cells, Clinical trials, Stromal cells

## Abstract

Mesenchymal stromal cells (MSCs), present in the stromal component of several tissues, include multipotent stem cells, progenitors, and differentiated cells. MSCs have quickly attracted considerable attention in the clinical field for their regenerative properties and their ability to promote tissue homeostasis following injury. In recent years, MSCs mainly isolated from bone marrow, adipose tissue, and umbilical cord—have been utilized in hundreds of clinical trials for the treatment of various diseases. However, in addition to some successes, MSC-based therapies have experienced several failures. The number of new trials with MSCs is exponentially growing; still, complete results are only available for a limited number of trials. This dearth does not help prevent potentially inefficacious and unnecessary clinical trials. Results from unsuccessful studies may be useful in planning new therapeutic approaches to improve clinical outcomes. In order to bolster critical analysis of trial results, we reviewed the state of art of MSC clinical trials that have been published in the last six years. Most of the 416 published trials evaluated MSCs’ effectiveness in treating cardiovascular diseases, GvHD, and brain and neurological disorders, although some trials sought to treat immune system diseases and wounds and to restore tissue. We also report some unorthodox clinical trials that include unusual studies.

**Graphical abstract:**

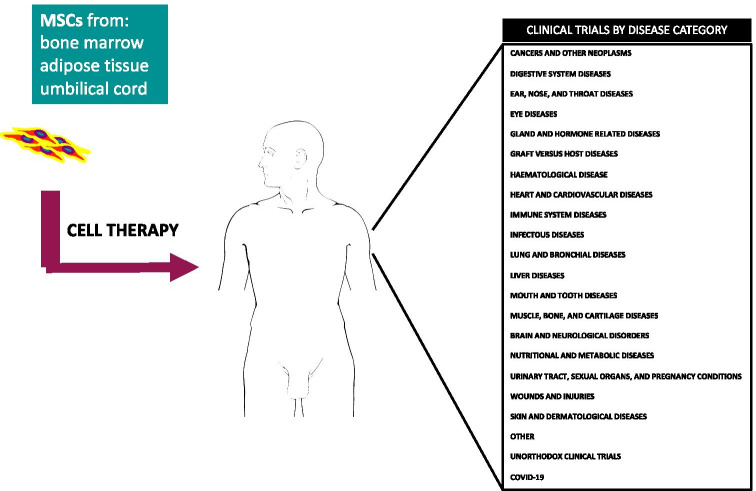

## Introduction

Mesenchymal stromal cells (MSCs), present in most stromal tissues, are a heterogeneous population containing multipotent stem cells, progenitors, and differentiated cells [1, 2]. MSCs are able to differentiate into mesodermal progeny, such as osteocytes, chondrocytes, adipocytes, and muscle cells [3]. MSCs were first characterized in 1967 by Friedenstein, who separated adherent clonogenic fibroblast-like colonies (colony-forming unit fibroblasts, or CFU-F) from bone marrow (BM). The cells originating from CFU-F colonies were distinct for their strong in vitro replication capacity and their ability to differentiate into osteocytes, chondrocytes, and adipocytes. In addition, when re-transplanted in vivo, CFU-F colonies were able to support bone marrow microenvironments [4].

In 1995, Hillard Lazarus performed a cell therapy with MSCs for the first time; since then, this approach has become one of the greatest clinically-experimental approaches in the world. Lazarus’s team reported a phase I trial evaluating the suitability of human bone marrow-derived progenitor stromal cells after ex vivo culture expansion and intravenous infusion in 23 patients with hematological malignancies [5].

Until 2006, the lack of homogeneous criteria for isolating and cultivating MSCs made it difficult to establish a reliable and reproducible application in the preclinical and clinical fields. This issue led the International Society for Cellular Therapy (ISCT) to recommend essential and objective criteria that are useful in characterizing the unique population of MSCs [6]:

- MSCs must be plastic-adherent when grown under standard conditions.
- The majority of the MSC population (≥ 95%) must be positive by flow cytometry for CD105, CD73, and CD90; must express low levels of MHC class I; and must be negative for MHC class II, CD11b, CD34, CD14, CD45, and CD31.
- MSCs must be able to differentiate in vitro in various tissues of mesodermal origin—such as osteocytes, adipocytes, and chondrocytes—under appropriate growth conditions.

## Mesenchymal Stromal Cells or Mesenchymal Stem Cells?

**Fig. 1:**
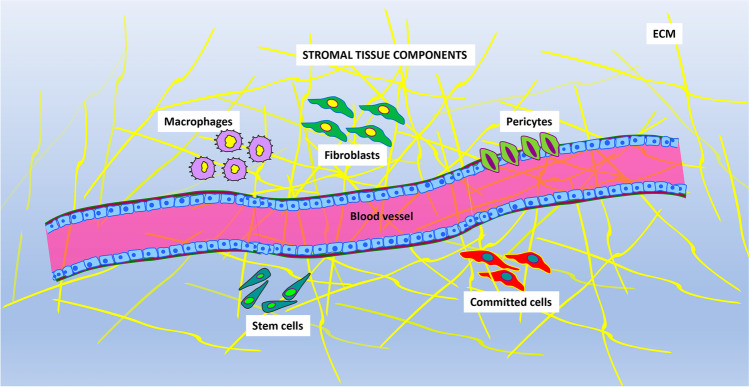
The picture shows the main components present in stromal tissues. Different cell phenotypes are intermingled with blood vessels and extracellular matrix (ECM) components. The principal cell types are: macrophages, fibroblasts, pericytes, committed cells and stem cells

Over the years, the use of the acronym ‘MSC’ has caused a great deal of confusion in the literature. The term “mesenchymal stem cell” was introduced by Caplan in the early 1990s [7], indicating that MSCs provide a production source of dermis, muscles, tendons, bones, cartilages, ligaments, connective tissue, and adipocytes. However, definitive data supporting the stemness of this heterogeneous cell population were not provided [8, 9]. It has been hypothesized that a stromal stem cell population may be present in the stromal component of the bone marrow microenvironment and in that of other tissues; thus, the term “mesenchymal stem cell” should be restricted to this population of mesenchymal cells (Fig. 1) [10]. Additionally, the ISCT has suggested that the plastic-adherent cells fluently defined as mesenchymal stem cells should be named ‘multi-potent mesenchymal stromal cells’. This statement inspired the scientific community to approve this nomenclature in all written documents and oral communications [11]. Although bone marrow represents the majority and primary origin of MSCs, several depots have been identified as alternative MSC reservoirs, including adipose tissue [12], umbilical cord blood [13], placenta [14], yellow ligament [15], and dental pulp [16].

In the human body, the impairment of MSC activities has huge repercussions on health, given their key role in tissue and organ homeostasis. These properties have been exploited in MSC-based cell therapy. Transplanted MSCs can efficiently focus on injured tissues and actively participate in tissue repair by secreting cytokines and growth factors that can restore tissue homeostasis, reducing local inflammation and differentiating into one or more of the cell types’ residents in the injured tissues [17, 18]. Moreover, MSCs decrease inflammation and increase cell proliferation during tissue regeneration via release of exosomes containing a variety of cargo, including mRNAs, proteins, and microRNAs [19, 20]. MSCs exhibit immunoregulatory properties by interacting with the innate immune system, showing both pro-inflammatory and anti-inflammatory activities. These unique properties make MSCs useful candidates for the treatment of various congenital and acquired diseases [21]. Many clinical trials have been conducted to evaluate the reliability and efficacy of cell therapy, and thousands of patients have received MSC transplants in order to treat different diseases: graft-versus-host disease (GvHD), malignant neoplasms, heart diseases, immune system diseases, and neurological disorders. An international panel of experts has established a reference plan to standardize MSC therapeutic treatments so as to avoid the spread of dubious stem cell therapy centers and the uncontrolled use and commercialization of unproven stem cell treatments. In this context, a methodological algorithm called DOSES has been proposed: D—Donor (i.e., autologous, allogeneic, or xenogeneic), O—Origin Tissue, S—Separation Method, E— Exhibited Characteristics (associated with cell behavior), S—Site of Delivery [2, 22, 23]. This standard may improve MSC treatments.

The current review describes the state of art of recent MSC clinical trials from 2015 to the present and highlights the limits and potentialities of cell therapy [24].

## MSC Features

**Fig. 2:**
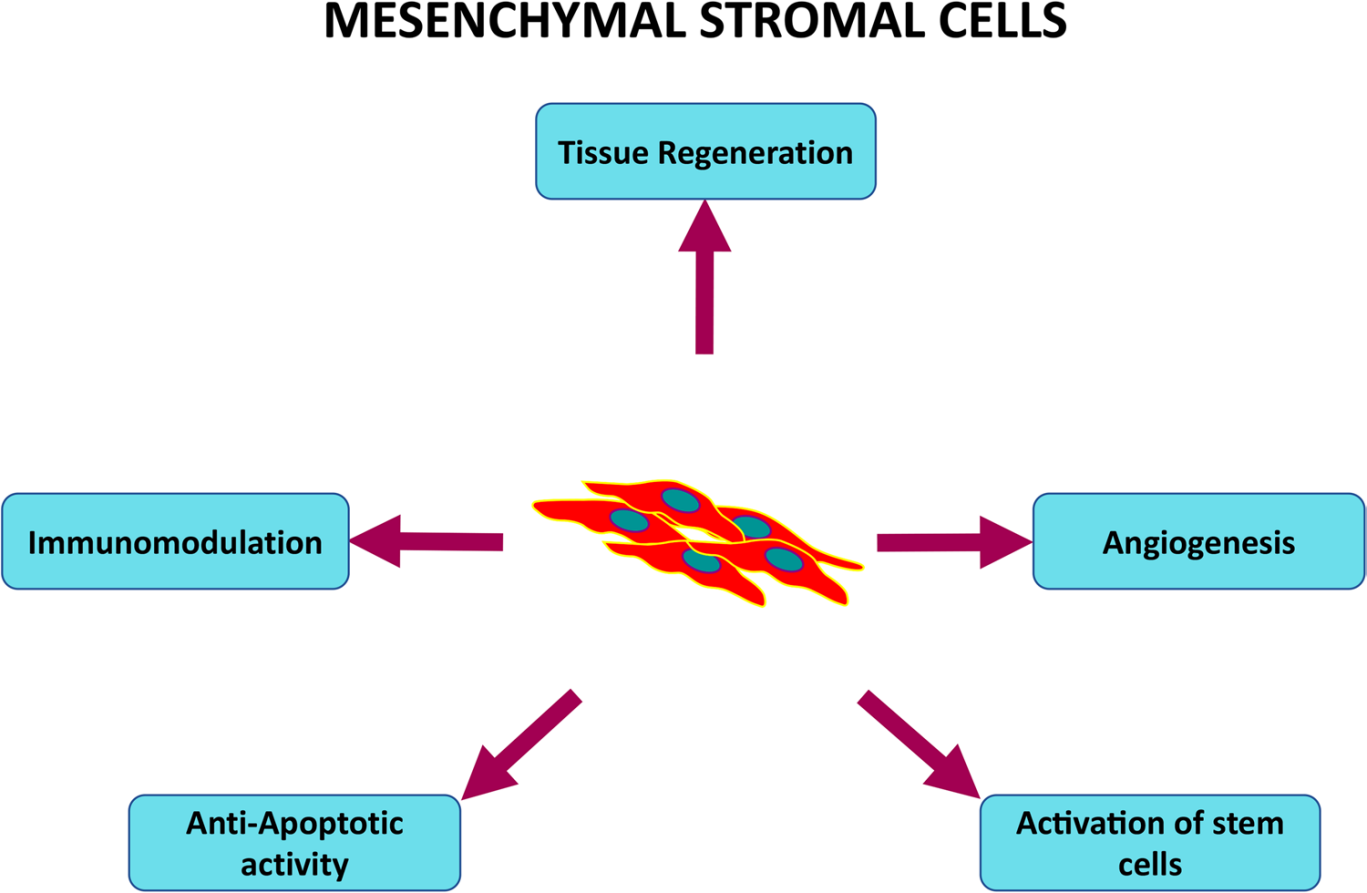
The summary of MSC mechanisms in cell therapy

The following paragraphs describe the main biological properties of MSCs, providing a better understanding of their therapeutic potential (Fig. 2).

## Population Heterogeneity

MSCs contain multipotent stem cell populations that can be committed to some specific cells lineages [25]. Several findings have shown a heterogeneity in the differentiation potential of these cells, as MSC cultures include stem cells with tri-, bi-, and uni-potential differentiation capabilities [26] [27, 28].

MSCs can be obtained from various fetal or adult tissues. In comparisons of MSCs isolated from adult sources with those from fetal tissues, the latter showed higher proliferation capacity, higher differentiation potential, and prolonged in vitro life span before onset of replicative senescence [29].

MSCs isolated from different adult tissues also showed biological heterogeneity. In particular, MSCs extracted from umbilical cord blood were unable to differentiate in adipocytes, while those originating from bone marrow showed a bias toward differentiation in osteocytes [30]. No clear correlations have been established between MSC properties and gender/age of donors [31], although some investigations have indicated a reduction in proliferation rate, life span, and differentiation potential in MSCs obtained from elderly donors [32]. These discrepancies suggest a probable environmental niche memory, which could be useful in isolating the most suitable tissue source for a given clinical application [30, 33].

## Secretome of MSCs

The MSC secretome has been described as an elaborate blend of bioactive molecules containing both a free fraction—composed mainly of growth factors, chemokines, cytokines, lipid intermediary molecules, cellular adhesion molecules, interleukins, and hormones—and a vesicular encapsulated fraction—formed by exosomes and microvesicles that are engaged to carry a variety of cargos, including RNAs (e.g., microRNAs), lipids, proteins, and DNA [34, 35]. These circulating factors are mainly involved in tissue regeneration and repair through paracrine effects that mediate cell-to-cell signaling. This cross-talk communication between the cells and the surrounding tissues plays a key role in immunomodulation, angiogenesis, anti-cancer, and tissue protection [36]. In the past decade researchers have turned a spotlight onto the importance of the MSC secretome both in cell transplant treatments and in cell-free therapies [37].

## Secretome Immunomodulatory Properties

MSC activities can modulate both adaptive and innate immune responses [38, 39]. For this reason, MSCs exhibit therapeutic potential in the treatment of immune system disorders [40, 41].

The inflammatory response acts through Toll-like receptors, which are activated following noxious stimuli; their activation counteracts pathogenic agents or stimuli from damaged tissues [42]. The activation of Toll-like pathways triggers immune responses based on the secretion of inflammatory molecules and phagocytosis. MSCs and stromal components are also activated to complete the immune response, characterized by high levels of TNF-α and IFN-γ [42, 43]. In particular, MSCs show two different polarization phenotypes, pro-inflammatory (MSC1) or anti-inflammatory (MSC2), that depend on Toll-like receptor (TLR3, TLR4) signaling.

The short-term stimulation of TLR4 on MSCs and the low levels of TNF-α and IFN-γ supports the establishment of a pro-inflammatory MSC1 phenotype, which increases T cell reactions via chemokines secretion, such as RANTES, MIP-1α MIP-1β, CXCL9, and CXCL10. These factors are registered in an inflammation area [44]. A sustained TLR3 stimulation of MSCs, associated with high levels of TNF-α and IFN-γ, triggers the MSC2 anti-inflammatory phenotype, which promotes the release of effector molecules that are capable of inhibiting cell-mediated and humoral activities of the immune system [45]. The anti-inflammatory properties of MSCs have been thoroughly investigated. For example, recent studies have indicated that MSCs, via soluble factors (IL-10, prostaglandin E2, IL-6, and TGF-β), are able to prevent dendritic cell-induced T cell activation and growth, thus blocking innate immune response and sustaining immune tolerance [46]. Other studies have proven that MSC-derived extracellular vesicles are able to modulate B lymphocytes’ proliferation and to promote the activity of regulatory B cells, which possess IL-10-associated immunosuppressive capacity [47]. Similarly, clinical applications and experimental models have demonstrated the immunosuppressive role of MSCs in modulating natural killer (NK) cell responses [48]. MSCs significantly decreased IL-2/15-induced proliferation of NK cells, their cytotoxicity, and the production of IFN-γ [49]. All these features are very important in improving the engraftment of bone marrow transplantation [50]. Macrophage activation is another aspect of the innate immune system [51]; in the inflammation area, MSCs control the polarization of macrophages in activated M2 macrophages, through the production of cyclooxygenase 2 and the expression of indolamine 2,3-dioxygenase [52]. In the absence of IL-6, MSCs polarize monocytes in activated M1 macrophages that, unlike M2, increment local inflammation by secreting pro-inflammatory cytokines (IFN-γ and TNF-α). Therefore, the interactions between MSCs and the innate immune system to balance pro- and anti-inflammatory responses are important to preserving tissue functionality and integrity.

## Secretome Angiogenic, Pro-survival, and Tissue Regeneration Properties

The paracrine function of MSCs represents one of the primary mechanisms supporting the effectiveness of MSC-based cell therapy. After transplantation, the great majority of MSCs persist in recipients’ organs for a short period of time (around 48 h) [53], and thus their beneficial effects cannot be ascribed to differentiation processes that replace damaged cells. Indeed, transplanted MSCs release a plethora of growth factors, chemokines, and cytokines able to perform angiogenic, immunomodulatory, antioxidative, and antiapoptotic effects during the initial days following MSC infusion [54, 55].

Various studies have demonstrated the role of MSCs in angiogenesis and arteriogenesis, useful in improving coronary heart disease, skin wound healing, and hindlimb ischemia [56–58]. Acting as intermediary of angiogenesis, MSCs release various growth factors, such as vascular endothelial growth factor (VEGF), fibroblast growth factors (FGFs), hepatocyte growth factor (HGF), stromal cell-derived factor 1 (SDF-1), and angiopoietin-1 (ANG-1). All these molecules are fundamental to endothelial cell growth and vascularization [59, 60]. MSCs are also involved in direct inhibiting apoptosis and promoting tissue regeneration by secreting insulin-like growth factor-I (IGFI), stanniocalcin-1 (STC1), B-cell lymphoma 2 (BCL-2), survivin, and granulocyte–macrophage colony-stimulating factor (GM-CSF). In a recent study, a mouse model of a chemically-injured cornea was used to demonstrate change in the expression of genes (*Bcl-2*, *Bax*, *P53*) associated with apoptosis. Transplantation of MSCs was able to mitigate the changes attenuating apoptosis [61].

Many pathological conditions (e.g., inflammation, cellular damage, and metabolism disorders) are associated with oxidative stress events that promote the formation of damaging reactive oxygen species. Different animal models have indicated a therapeutic antioxidant property of MSCs via free radicals scavenging, stimulation of physiological antioxidant defenses, modification of mitochondrial bioenergetics, and mitochondria transferring to injured cells through tunneling nanotubes [62–65]. Several studies have demonstrated that MSCs are also highly resistant to oxidative stress induced by ionizing radiation [66]. Globally speaking, MSCs can modulate their cytoprotective and anti‐inflammatory features by setting the redox environment and oxidative stress.

## MSC-based Therapy: A Recent Overview of Clinical Trials

In the past ten years, stem cell therapy has represented a revolutionary tool for regenerative medicine, and MSCs have been a valuable therapeutic approach in the treatment of several diseases, providing significant benefits in tissue repair strategies thanks to various advantages such as autologous transplantation, safety around cellular division, and lack of teratoma onset [67–69].

**Fig. 3:**
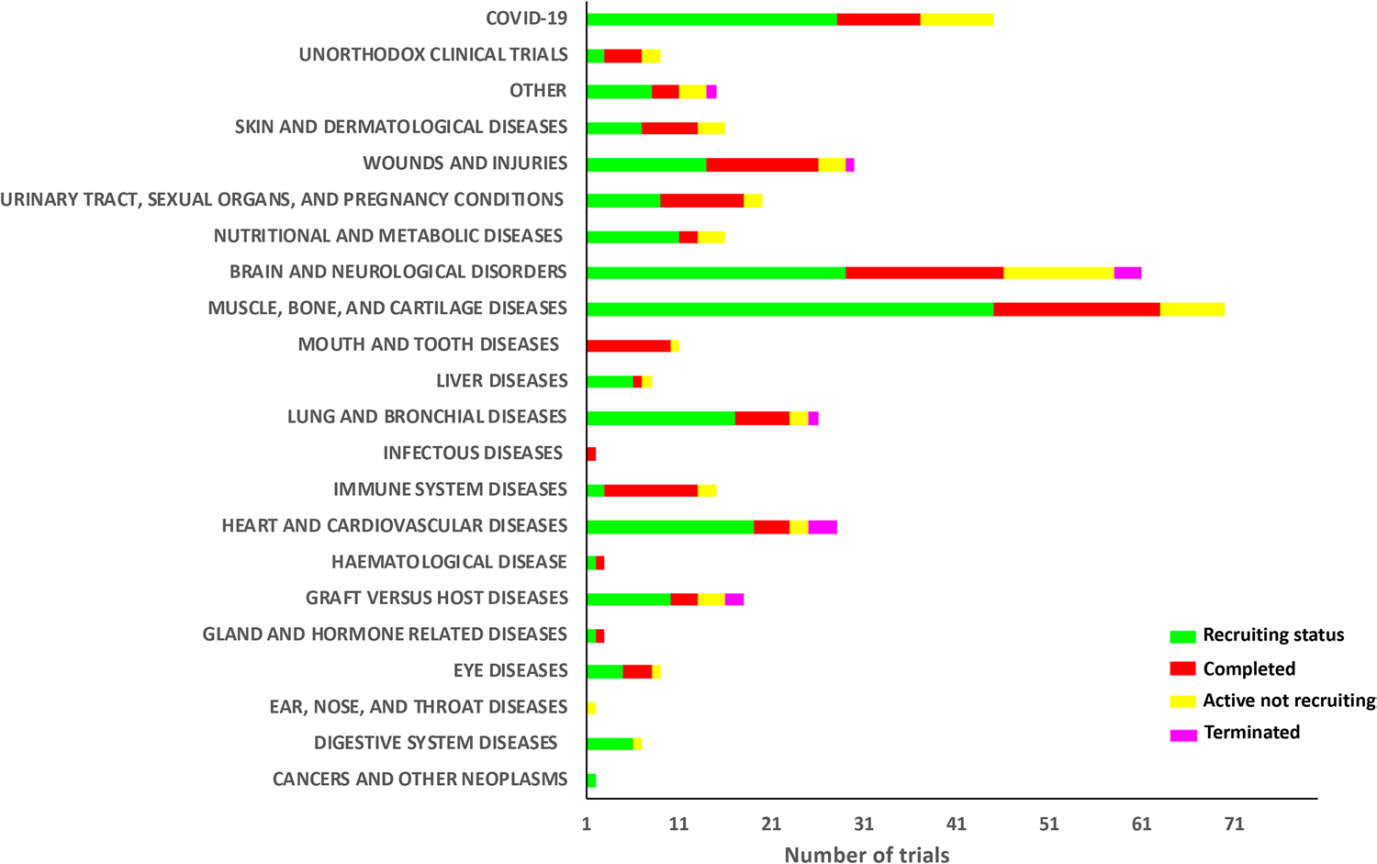
MSC clinical trials classified by disease categories were also subdivided by progress status: in green, “Recruiting Status”; in red, “Completed”; in yellow, “Active not recruiting”; in magenta, “Terminated”. The 22 shown categories account for > 90% of the trials reported on clinicaltrials.gov from 2015 to present

**Fig. 4:**
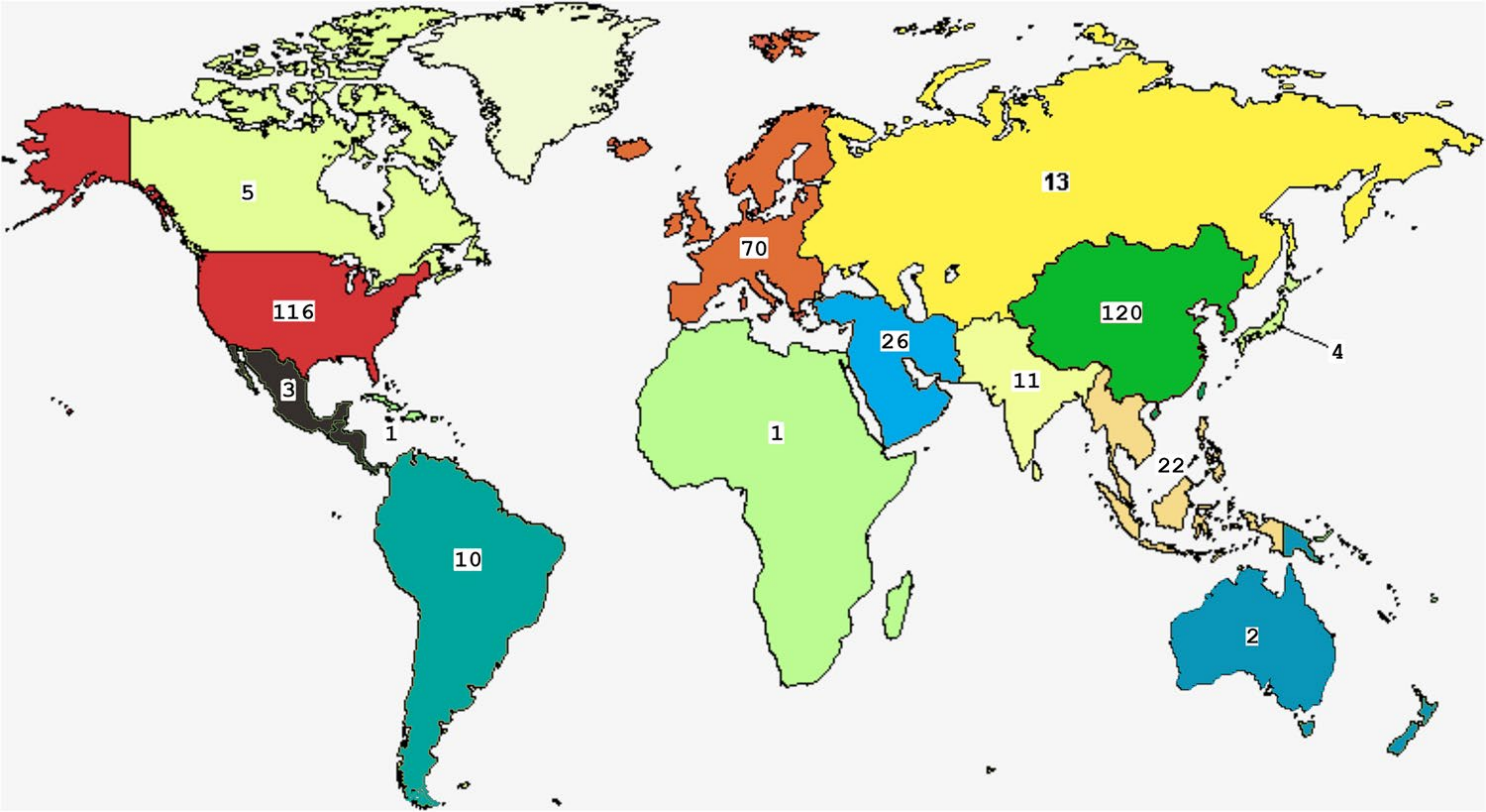
Geographic distribution of MSC clinical trials in the world. The world map was obtained from clinicaltrials.gov

From 2015 to present, 416 clinical trials based on the use of MSCs have been implemented to treat various pathologies, according to the US National Institute of Health–ClinicalTrials database (http://clinicaltrials.gov). At present, of these trials, 55 are *Active non-recruiting*, 233 studies are *Recruiting*, 117 are *Completed*, and 11 trials are *Terminated* (ended prematurely) (Fig. 3). The great majority of trials have been carried out in the U.S.A. and China, but several investigations have been performed in other countries too (Fig. 4).

The following sections describe the most significant trials, grouped by type of target diseases. We focused our attention on completed trials, primarily Phase II–III trials. In the text, the studies are reported with the corresponding clinicaltrials.gov identifier (NCT).

As a reminder for readers, clinical trials are divided in four phases.

*Phase I:* A small group of healthy volunteers (20–100 individuals) undergoes treatment for several months to test the safety and dosage of an experimental procedure.

*Phase II*: A higher number of enrolled patients is required (up to several hundred) to evaluate efficacy and side effects of the treatment.

*Phase III*: Effectiveness and safety are further evaluated, sometimes in combination with other well-known pharmaceutical treatments. Regulatory medical agencies approve the treatment if safety, efficacy, and reduced adverse reactions of the treatment are proven during this phase.

*Phase IV*: With large and multiple populations, the treatment is monitored for a long time period. The analysis of treatment continues after its release on the market to check safety and efficacy.

## Cardiovascular Diseases

According to the World Health Organization, cardiovascular disease is a group of disorders of heart and blood vessels including hypertension, coronary heart disease, peripheral vascular disease, and myocardial infarction. Cardiovascular diseases represent one of the primary causes of death and disability. After myocardial infarction, cardiomyocytes die and their function is lost with subsequent ventricular remodeling, inflammatory response, and fibrous scar formation that lead to heart failure [70]. In addition to drug treatments and heart transplantation [71, 72], the use of MSCs has appeared as an encouraging cell therapy for heart disease in the past decade [18, 73, 74].

An interesting completed study in phase I (NCT02509156) sought to evaluate the safety and feasibility of transendocardial injection of bone marrow allogenic MSCs (allo-MSCs) in cancer survivors with anthracycline-induced cardiomyopathy (AIC). The secondary objectives were to determine the effects of allo-MSCs’ administration on left ventricular function and functional status from baseline to 6 months and from baseline to 12 months after treatment. In this study, 37 patients met the eligibility criteria, one of which was that patients must be clinically free of cancer for at least two years with a ≤ 30% estimated five-year risk of recurrence. This study, named SENECA (StEm cell iNjECtion in cAncer survivors), offers a promising new approach to repairing damaged myocardium. The first six subjects participated in an open-label, lead-in phase trial and received 100 million allo-MSCs; the remaining 30 patients will be randomized 1:1 to receive allo-MSCs or vehicle via 20 transendocardial injections. All endpoints are/will be assessed at 6- and 12-months post-treatment. The approach of the SENECA study represents the first clinical trial for treating AIC through direct cardiac injection of cells. Preliminary results and further investigations have been published [75] [76]. The researchers involved in this trial concluded that transendocardial cell administration appears safe and feasible, although the phase I study was small and not powered or designed to assess efficacy; larger phase II and III trials aimed at assessing efficacy are needed.

In another clinical trial (NCT02501811), the same research group proposed an investigation named CONCERT-HF (Combination of Mesenchymal and c-kit^+^ Cardiac Stem Cells As Regenerative Therapy for Heart Failure). This trial aimed to evaluate the feasibility, safety, and efficacy of transendocardial administration of autologous MSCs (target dose: 150 million) and cardiac stem cells (c-kit ^+^) (target dose: 5 million), alone and in combination, in patients with heart failure caused by chronic ischemic cardiomyopathy. CONCERT-HF was designed as a randomized, double-blind, placebo-controlled trial and enrolled 162 patients—18 in a safety lead-in phase (Phase I) and 144 in the main trial (Phase II). Phase I is completed, and Phase II is currently randomizing patients from 7 centers across the United States. Each participant will have regular follow-up visits after one day, one week, one month, three months, six months, and 12 months. CONCERT-HF will examine whether the cell administration alleviates left ventricular remodeling and dysfunction, reduces scar size, improves quality of life, or augments functional capacity in this population sample. Preliminary data encourage the use of CONCERT-HF for its potential therapeutic utility [77]. Final results are still not available.

The remaining clinical trials for cardiovascular disease in recruiting status (Phase I or Phase II) offer some other options: PERISCOPE (Pericardial Matrix With Mesenchymal Stem Cells for the Treatment of Patients With Infarcted Myocardial Tissue–NCT03798353), MESAMI2 (Administration of Mesenchymal Stem Cells in Patients With Chronic Ischemic Cardiomyopathy–NCT02462330), and AMASCIS-02 (Allogeneic Adipose Tissue-derived Mesenchymal Stem Cells in Ischemic Stroke–NCT04280003). These trials use MSCs isolated from allogenic umbilical cord, Wharton’s jelly, or autologous bone marrow to validate the safety and potential benefits of cell therapy performed with intramyocardial or intravenous injections.

In another trial (NCT02635464), human umbilical cord-derived MSCs were loaded into a collagen scaffold and injected into the infarct region to determine whether cell-laden hydrogel treatment is safe and feasible for patients with chronic ischemic cardiomyopathy. Among 115 eligible patients with chronic ischemic heart disease, 50 patients with left ventricular ejection fraction of 45% or less were selected to receive elective coronary artery bypass grafting and were additionally randomized to cell-plus-collagen treatment (collagen/cell group), cell treatment alone (cell group), or a control group. Sixty-five patients were excluded due to severe comorbidities or unwillingness to participate. Forty-five participants (88%) completed the study; the last patient completed their 12-month follow-up in August 2019. The encouraging data provides the first step to supporting clinical use of collagen hydrogel as support for cell delivery [78].

## Graft Versus Host Disease

Allogeneic hematopoietic stem cell transplantation appears to be the most successful strategy to treat hematological and autoimmune diseases [79, 80]. Nevertheless, graft versus host disease (GvHD) represents one of the major elements limiting the success of this therapeutic approach, influencing the survival percentage of grafted patients [81]. GvHD is also frequent after host tissue and solid organ transplantation (lung, skin, liver, and digestive tract) [82, 83]. According to different pathologic parameters, GvHD has been clinically classified as acute (aGvHD) or chronic (cGvHD). aGvHD is identified by T helper cells 1 activation, while cGvHD is principally attributed to the T helper cells 2 response [80]. Steroid therapy is an early approach for treating aGvHD [84]; nevertheless, in many cases (30–50%), aGvHD is not controlled with steroid treatment and requires additional therapeutic strategies [85]. In this context, MSCs, with their immunomodulation abilities, can play a pivotal role in the therapeutic approach to counteracting aGvHD that is resistant to steroid treatments.

Bloor et al. enrolled sixteen patients with steroid-resistant aGvHD (SR-aGvHD) in a phase I, open-label clinical trial (NCT02923375).The subjects were divided in two groups (cohort A and cohort B) and received intravenous administration of iPSC-derived MSCs on days 0 and 7. Cohort A received a dose of 1 million cells/kg, up to a maximum of 100 million cells, while cohort B received a dose of 2 million cells/kg, up to a maximum of 200 million cells. The iPSCs derived from patients’ fibroblasts offered a great advantage, as they accelerate the production of a fruitfully unlimited amount of MSCs from a single blood donation, overcoming the limits associated with production of donor-originated MSCs. The principal aim of Bloor et al.’s investigation was to estimate the safety and tolerability of the approach and to assess its efficacy by evaluating the percentage of candidates that displayed a complete response (53.3%), an overall response (86.7%), and overall survival (86.7%) by day 100 [86]. As described by the authors, the procedures are safe and well-tolerated, without significant adverse events, and thus could be also investigated in relation to other inflammatory and immune-mediated diseases.

Kurtzberg et al. described a phase III prospective study (NCT02336230) to treat 54 pediatric subjects (male and female) between the ages of 2 months and 17 years who were suffering from corticosteroid-resistant aGvHD. Patients were treated with an intravenous MSC injection (Remestemcel-L) at a dose of 2 × 10^6^ MSCs/kg twice per week for 4 consecutive weeks. The treatment significantly boosted overall response rate (OR) by day 28 compared to the control. The statistically significant OR (70.4%) persisted through day 100 and included an enhancement in complete response from 29.6% at day 28 to 44.4% at day 100. Overall survival was 74.1% at day 100 and 68.5% at day 180 [87].The authors state that Remestemcel-L therapy is tolerated, safe, and non-toxic, representing a valid treatment for pediatric patients suffering from steroid-resistant aGvHD.

## Brain and Neurological Disorders

MSC-based therapies also appear as a promising new treatment for several brain and neurological diseases. The neurotrophic, migratory, immunosuppressive, and tissue-regenerative properties of MSCs could be useful for treating neural injuries and neuropathologies with an inflammatory etiology [88–90]. In recent years, various clinical investigations have reported the therapeutic advantages of MSC-based cell therapy in treating stroke (NCT02378974, NCT03371329), multi-system atrophy (NCT03265444), multiple sclerosis (NCT02495766, NCT02403947), amyotrophic lateral sclerosis (NCT03280056), Parkinson’s disease (NCT02611167, NCT03684122), Alzheimer’s Disease (NCT03117738), and chronic spinal cord injury (NCT02152657, NCT02981576, NCT02570932).

Petrou et al. described in their study (NCT02166021) the health effects of autologous MSC transplantation in multiple sclerosis patients. They evaluated the optimal procedure of MSC administration, its safety, and its clinical efficacy [91]. Between 2015 and 2018, 48 patients with active and progressive multiple sclerosis were enrolled. Patients were randomized into 3 different groups: intrathecal, intravenous (in both cases using 1 × 10^6^/kg autologous MSCs), and sham injections. Six months later, half of the patients that had received MSCs via intrathecal and intravenous injection were submitted to a second cell treatment, while the remaining half received a placebo. The authors claim that the cell treatments were well tolerated and enabled short-term healthy effects concerning the primary end points, particularly in patients with active disease. Furthermore, in an evaluation of different disease parameters, intrathecal injection was more efficacious than intravenous administration, although a phase III trial is needed to confirm these results.

In a similar investigation, a clinical trial is being performed to explore the efficacy and safety of MSC-based therapy for treating multiple sclerosis (NCT02239393). The study, named MESEMS (MEsenchymal StEm cells for Multiple Sclerosis), has been planned to connect partially independent clinical trials, follow uniformed protocols, and share some key centralized procedures, such as data analysis and collection [92]. The data are still being evaluated.

## Muscle, Bone, and Cartilage Diseases

The ability of MSCs to differentiate into osteocytes, chondrocytes, adipocytes, muscle cells, and other tissues of mesenchymal origin makes them an ideal candidate in the treatment of bone and cartilage diseases. Osteoarthritis is the most common form of knee arthritis; it is a degenerative disease that occurs mainly in individuals older than 50 years, although it may be also present in young people. In knee osteoarthritis, cartilage articulation a slow, progressive erosion, causing bone rub and promoting painful bone spurs [93]. For these reasons, knee osteoarthritis is one of the most investigated bone and cartilage disorders in MSC-based clinical studies (NCT02580695, NCT04326985, NCT02351011, NCT03337243, NCT02958267, NCT03509025, NCT04037345, NCT02674399, NCT03000712, NCT03990805, NCT01879046).

JOINTSTEM (NCT02658344) is a clinical trial using autologous adipose-derived MSCs. The aim of this study was to promote cartilage growth and regeneration within six months in knees of patients with osteoarthritis. The study was a phase IIb prospective, double-blinded, randomized trial. The MSCs (1 × 10^8^ cells in 3 mL of saline solution) were intra-articularly injected in 12 patients, and outcomes were compared with 12 other patients (control group) who were administered a saline solution. According to the authors, the intra‐articular injection of autologous adipose MSCs provided functional progress and pain reduction in treated patients, without side effects [94]. Nevertheless, long‐term studies should be carried, as 6 months is a short time period for evaluating clinical efficacy and structural outcomes. Moreover, large sample cohorts are necessary.

In another trial, Zhao et al. used allogeneic adipose-derived MSCs in knee osteoarthritis patients. Eighteen patients participated a phase I/IIa clinical trial (NCT02641860). All individuals were randomized into three groups (six patients each) and received a low-dose (1.0 × 10^7^ cells), mid-dose (2.0 × 10^7^), or high-dose (5.0 × 10^7^) intra-articular injections of cells [95]. Preliminary data showed functional progress and pain reduction in treated patients. Here, too, larger patient cohorts and long-term follow-up are required.

## Lung and Bronchial Diseases

Pawel and Silvestri used PNEUMOSTEM^®^ as a cellular therapy product for the prevention of bronchopulmonary dysplasia in premature infants at high risk of death. The study (NCT02381366) is an open-label, single-center, dose escalation investigation, consisting of patient injection with ex vivo cultured allogeneic, unrelated, human umbilical cord blood-derived MSCs. The experimental procedure consisted of two cellular doses: 6 of 12 patients received dose A (10^6^cells/kg), while the remaining received dose B (20^6^cells/kg) by intratracheal administration. All patients tolerated the administration, with no toxicities during the first monitoring period (72 h) and no side effects during the 84 subsequent days [96]. However, the cohort was too small to evaluate real benefit; a larger randomized study is necessary to establish the effectiveness of such a therapy.

Pneumoconiosis is a lung disease that occurs due to the inhalation of fine particles, such as silica. (For this reason, the disease is commonly called silicosis.) Currently, there is no effective drug treatment improving respiratory function and reversing the progression of pulmonary fibrosis. At present, one trial (NCT02668068) analyzes and evaluates the safety and effectiveness of clinical-grade umbilical cord MSC transplantation for pneumoconiosis treatment in combination with whole-lung lavage, a conventional therapy. Eighty participants have been enrolled, who will receive 10^6^cells/kg/person that will be injected after whole-lung lavage. The trial is still ongoing.

## Wounds and Restorations

Foot ulcers are a common consequence in diabetic patients as a result of skin breakdown. Infected ulcers could cause limb amputation if they are neglected. A non-healing minor amputation frequently precedes a major amputation that will have a huge impact on the function and quality of life of the patient.

Lonardi et al. evaluated the healthy benefits of autologous adipose MSC injection at the amputation stump of diabetic patients who had minor lower limb amputation. In this randomized controlled trial (NCT03276312), 114 patients undergoing a minor lower limb amputation were randomized to standard care or to cell treatment, called Lipogems®. This latter treatment is based on a minimum quantity of autologous lipoaspirates that were used, with a minimal manipulation, for patient injection. The rationale behind this procedure is the presence of unpurified MSCs in lipoaspirates. By evaluating the healing rate and the time after the minor amputation, the authors found that Lipogems® improved the healing rate of treated feet. The approach was safe, and technical success was obtained in all cases. Additionally, skin tropism and pain scale were improved [97].

Moon et al. examined the potential of allogenic adipose-derived MSCs in treating diabetic foot ulcers. Fifty-nine patients were randomized into two groups (n = 30 and n = 29) for MSC administration or polyurethane film (control), respectively. The cells embedded within the film or the film alone were applied on diabetic wounds weekly (NCT02619877), and these wounds were assessed for 12 weeks. At two months, entire wound closure was obtained in 73% of patients in the treatment group and in 47% of patients in the control group; at three months, total wound closure was 82% in the treatment group and 53% in the control group [98]. No serious collateral effects were found to be associated with cell therapy. Thus, allogeneic MSCs might be a valid and safe approach to treat diabetic foot ulcers.

A Phase I study (NCT02589119) with 15 adult patients aged 18 years and over with cryptoglandular fistulas and used a single dose of 20 million MSC-coated fistula plugs (Gore Fistula Plug). Patients first underwent standard adjuvant therapy, which included drainage of the infection and placement of a draining seton. A month and a half later, the seton was replaced with an MSC-coated fistula plug. The subjects were subsequently followed up with for 24 months for response and closure of the fistula. As yet, no study results have been published.

## Immune System Diseases

Systemic sclerosis is a progressive autoimmune disease that results in disability due to diffuse fibrosis and vascular abnormalities in the skin, joints, and internal organs.

Park et al. performed an open-label study in early phase I (NCT03060551) to investigate the efficacy and safety of an autologous fat tissue-derived stromal vascular fraction injected into systemic sclerosis patients. Twenty patients with hand disability received an average of 3.61 × 10^6^ MSCs in each finger. A 24-month follow-up had been planned for every patient. According to the results, autologous adipose tissue-derived MSC injection into patients is well tolerated. The trial indicated clinical efficacies of the proposed treatment, as it promoted improvement of skin fibrosis, quality of life, and attenuation of digital ulcers [99].

Autologous adipose-derived MSCs were also used to treat autoimmune refractory epilepsy. The study was registered with trial identifier NCT03676569. In six patients, intrathecal injection of autologous MSCs was repeated 3 times every 3 months. All parameters and antiepileptic effects were monitored for 12 months. All patients showed clinical improvement after injections. In some cases, transient mild side effects were observed, including increased body temperature, pain due to liposuction, and slight growth of epileptic episodes. After treatment, patients showed improved social functioning and intellectual performance [100].

Systemic lupus erythematosus is an autoimmune disease characterized by the production of auto-antibodies against cellular nucleus. Although approximately 50% of lupus patients have lupus nephritis, there is no therapy for this complication. For lupus treatment, three different studies are included on the clinical trials website as evaluating the safety and effectiveness of MSCs obtained from umbilical cords (NCT03171194), olfactory mucosa (NCT04184258), and allogenic bone marrow (NCT03174587). As yet, no study results have been published.

## Unorthodox Clinical Trials

The versatility of MSC-based therapy has paved the way to their application in odd trials, some of which completely lack scientific meaning. Others may have therapeutic effectiveness, even if the rationale behind them is not completely understood.

Mattei et al. proposed a clinical trial (NCT02622464) to treat patients suffering from scarred vocal folds. Scarred vocal folds are characterized by anomalous scar tissue present in the vibrating layer of the vocal folds, causing voice problems. The study considered autologous adipose tissue-derived stromal vascular fraction (by lipoaspirates) as a therapeutic candidate for treating scarred vocal folds, given the angiogenic, immunomodulatory, anti-inflammatory, and regenerative properties of stromal cells. This study, named CELLCORDES, was a prospective, open-label, single-arm, single-center, nonrandomized controlled trial. Eight patients with severe dysphonia due to vocal fold scarring were treated with stromal vascular fraction in situ (into 1 or 2 vocal folds) during laryngoscopy. The results indicate feasibility and tolerability of cell injection, but further studies using randomized clinical trials with an adequate number of patients are needed to evaluate the treatment effectiveness [101].

Jieming et al. at Ruijin Hospital in Shanghai, China, set up a clinical study (NCT04213248) to evaluate the safety and tolerance of aerosol inhalation of the exosomes derived from allogenic adipose MSCs in the treatment of severe lung diseases (including severe lung infection, acute respiratory distress syndrome, and chronic obstructive pulmonary disease). Their experimental plan is based on studies that have demonstrated the ability of MSCs and exosomes to significantly reduce lung inflammation and other lung injuries. Consequently, they argue that exosomes (containing cytokines, growth factors, signaling lipids, mRNAs, and regulatory miRNAs) may exhibit therapeutic effect comparable to MSC-based cell therapy. Results are still lacking.

In the clinical trial registered as NCT02742857, investigators aimed to establish a reversal of brain death using intrathecal injection of bioactive peptides and MSCs associated with laser stimulation of transcranial IV and median nerve. The research, which enrolled 20 patients, is completed, but no results have been provided.

One more unusual study (NCT04213248) is ongoing at the Zhongshan Ophthalmic Center at Sun Yat-sen University in China. The investigators seek to determine whether umbilical MSC-derived exosomes could alleviate dry eye symptoms in patients with cGvHD, as 60–90% of such patients are affected by dry eye symptoms, which may seriously affect their life quality. The effectiveness of exosome treatment will be evaluated by measuring the ocular surface index score. Other analyses will measure the tear secretion amount, the tear break time, the ocular redness, the tear meniscus, and the visual acuity. Approximately 27 subjects will be recruited. The treatment group will receive artificial tears for 2 weeks to normalize the baseline, followed by administration of exosomes at 10ug/drop, four times a day, for 14 days. The follow-up visit will be at 12 weeks post-treatment, where the progression of dry eye will be evaluated.

## COVID-19

On March 1st, 2020, the World Health Organization declared COVID-19 a pandemic infectious disease, given the thousands of coronavirus cases in over 110 countries and territories around the world. In recent months, more different scientific approaches have been proposed for treating coronavirus-affected patients. A growing number of MSC-based cellular therapies for the treatment of COVID-19 (NCT04491240, NCT04492501, NCT04573270, NCT04535856, NCT04355728, NCT04276987, NCT04713878) is seen on the clinical trials website. The rationale behind this choice is to exploit the anti-inflammatory and immunomodulatory properties of MSCs in order to reduce the damage caused by cytokine storm to tissues and organs, which are responsible for the onset of pneumonia, acute respiratory distress syndrome, and multi-organ failure. In the general hospital of Wuhan, China, where the pandemic started, Shi et al. performed a phase II trial (NCT04288102) to ascertain the efficacy and safety of human umbilical cord MSCs in treating severe COVID-19 patients with impairment of lung functions. The trial, which was randomized, double-blind, and placebo-controlled, recruited 101 patients with lung damage. Each of them received either MSCs (4 × 10^7^ cells per infusion) or a placebo on days 0, 3, and 6. Compared to the placebo, MSC injection by intravenous transfusion was found to significantly reduce the proportions of solid component lesion volume at day 28. The adverse effects were similar in the two experimental groups. According to the authors, the results suggest that MSCs represent a therapeutic approach that is safe and potentially effective for COVID-19 patients with lung damage [102]. Therefore, the authors are looking to perform a phase III trial to estimate the positive effects in terms of reducing mortality and preventing long-term pulmonary disability.

## Conclusions

We reviewed the state of art of MSC clinical trials—with MSCs isolated from bone marrow, umbilical cord, and adipose depots—conducted in the last six years (2015 to present). Most of these studies have analyzed the effectiveness of such cell therapies for the treatment of cardiovascular diseases, GvHD, and brain and neurological disorders, but there are also trials analyzing its use in treatment of immune system diseases and wounds and tissue restoration.

Although the number of new trials is exponentially growing, the results (positive or negative) are only published in a few completed clinical trials. This deficiency should be addressed, since lack of data cannot help prevent potentially inefficacious pleonastic clinical trials. Moreover, data on unsuccessful studies could pave the way to setting up new therapeutic strategies to improve clinical outcomes. Negative results may also derive from clinical trials with a very limited number of enrolled patients; thus, this approach should be highly discouraged. Some indications about how to standardize trials using MSCs were published several years ago, but these recommendations are still disregarded [103]. Donor variance, in vitro expansion, immunogenicity, senescence, and cryopreservation are key factors that can negatively affect the validity of MSC-based therapy. All these issues should be carefully addressed in order to provide reliable, reproducible, and effective therapies based on MSCs.

## Data Availability

N/A

## Code availability

N/A

## References

[1] Bianco P, Robey PG, Simmons PJ (2008). Mesenchymal stem cells: Revisiting history, concepts, and assays. Cell Stem Cell.

[2] Pittenger MF, Discher DE, Peault BM, Phinney DG, Hare JM, Caplan AI (2019). Mesenchymal stem cell perspective: Cell biology to clinical progress. NPJ Regenerative Medicine.

[3] Ciuffreda MC, Malpasso G, Musaro P, Turco V, Gnecchi M (2016). Protocols for in vitro differentiation of human mesenchymal stem cells into Osteogenic, Chondrogenic and Adipogenic Lineages. Methods in Molecular Biology.

[4] Friedenstein AJ, Petrakova KV, Kurolesova AI, Frolova GP (1968). Heterotopic of bone marrow. Analysis of precursor cells for osteogenic and hematopoietic tissues. Transplantation.

[5] Lazarus HM, Haynesworth SE, Gerson SL, Rosenthal NS, Caplan AI (1995). Ex-Vivo expansion and subsequent infusion of human bone-marrow-derived stromal progenitor cells (Mesenchymal Progenitor Cells)—implications for therapeutic use. Bone Marrow Transplantation.

[6] Dominici M, Le Blanc K, Mueller I, Slaper-Cortenbach I, Marini F, Krause D, Deans R, Keating A, Prockop D, Horwitz E (2006). Minimal criteria for defining multipotent mesenchymal stromal cells. The international society for cellular therapy position statement. Cytotherapy.

[7] Caplan AI (1991). Mesenchymal stem cells. Journal of Orthopaedic Research.

[8] Bianco P (2014). "Mesenchymal" stem cells. Annual Review of Cell and Developmental Biology.

[9] Keating A (2006). Mesenchymal stromal cells. Current Opinion in Hematology.

[10] Horwitz EM, Andreeff M, Frassoni C (2007). Mesenchymal stromal cell. Biology of Blood and Marrow Transplantation.

[11] Horwitz EM, Le Blanc K, Dominici M, Mueller I, Slaper-Cortenbach I, Marini FC, Deans RJ, Krause DS, Keating A, T International Society for Cellular (2005). Clarification of the nomenclature for MSC: The international society for cellular therapy position statement. Cytotherapy.

[12] Heidari B, Shirazi A, Akhondi MM, Hassanpour H, Behzadi B, Naderi MM, Sarvari A, Borjian S (2013). Comparison of proliferative and multilineage differentiation potential of sheep mesenchymal stem cells derived from bone marrow, liver, and adipose tissue. Avicenna Journal of Medical Biotechnology.

[13] Romanov YA, Svintsitskaya VA, Smirnov VN (2003). Searching for alternative sources of postnatal human mesenchymal stem cells: Candidate MSC-like cells from umbilical cord. Stem Cells.

[14] Papait A, Vertua E, Magatti M, Ceccariglia S, De Munari S, Silini AR, Sheleg M, Ofir R, Parolini O (2020). Mesenchymal stromal cells from fetal and maternal placenta possess key similarities and differences: Potential implications for their applications in regenerative medicine. Cells.

[15] Chen YT, Wei JD, Wang JP, Lee HH, Chiang ER, Lai HC, Chen LL, Lee YT, Tsai CC, Liu CL, Hung SC (2011). Isolation of mesenchymal stem cells from human ligamentum flavum implicating etiology of ligamentum flavum hypertrophy. Spine.

[16] Ledesma-Martinez E, Mendoza-Nunez VM, Santiago-Osorio E (2016). Mesenchymal stem cells derived from dental pulp: A review. Stem Cells International.

[17] Dimarino AM, Caplan AI, Bonfield TL (2013). Mesenchymal stem cells in tissue repair. Frontiers in Immunology.

[18] Guo Y, Yu Y, Hu S, Chen Y, Shen Z (2020). The therapeutic potential of mesenchymal stem cells for cardiovascular diseases. Cell Death and Disease.

[19] Lee C, Mitsialis SA, Aslam M, Vitali SH, Vergadi E, Konstantinou G, Sdrimas K, Fernandez-Gonzalez A, Kourembanas S (2012). Exosomes mediate the cytoprotective action of mesenchymal stromal cells on hypoxia-induced pulmonary hypertension. Circulation.

[20] Zhang B, Yin Y, Lai RC, Tan SS, Choo AB, Lim SK (2014). Mesenchymal stem cells secrete immunologically active exosomes. Stem Cells and Development.

[21] Castro-Manrreza ME, Montesinos JJ (2015). Immunoregulation by mesenchymal stem cells: Biological aspects and clinical applications. Journal of Immunology Research.

[22] Murray I. R., Chahla J., Safran M. R., Krych A. J., Saris AI Caplan D. B. F., LaPrade R. F., G Cell Therapies Communication Expert (2019) International expert consensus on a cell therapy communication tool: DOSES Journal of Bone and Joint Surgery. American Volume *101*, 904–911. 10.2106/JBJS.18.00915 PMC7292498 31094982

[23] Rodeo S. A. (2019). A Call for Standardization in Cell Therapy Studies: Commentary on an article by Iain R. Murray, BMedSci(Hons), MRCS, MFSEM, PhD, et al.: "International Expert Consensus on a Cell Therapy Communication Tool: DOSES". *J Bone Joint Surg Am 101*, e47. 10.2106/JBJS.19.00189 31094994

[24] Squillaro T, Peluso G, Galderisi U (2016). Clinical trials with mesenchymal stem cells: An update. Cell Transplantation.

[25] Muraglia A, Cancedda R, Quarto R (2000). Clonal mesenchymal progenitors from human bone marrow differentiate in vitro according to a hierarchical model. Journal of Cell Science.

[26] Okamoto T, Aoyama T, Nakayama T, Nakamata T, Hosaka T, Nishijo K, Nakamura T, Kiyono T, Toguchida J (2002). Clonal heterogeneity in differentiation potential of immortalized human mesenchymal stem cells. Biochemical and Biophysical Research Communications.

[27] Alessio N, Acar MB, Demirsoy IH, Squillaro T, Siniscalco D, Bernardo GD, Peluso G, Ozcan S, Galderisi U (2020). Obesity is associated with senescence of mesenchymal stromal cells derived from bone marrow, subcutaneous and visceral fat of young mice. Aging (Albany NY).

[28] Pochampally R (2008). Colony forming unit assays for MSCs. Methods in Molecular Biology.

[29] Hass R, Kasper C, Bohm S, Jacobs R (2011). Different populations and sources of human mesenchymal stem cells (MSC): A comparison of adult and neonatal tissue-derived MSC. Cell Communication and Signaling: CCS.

[30] Kern S, Eichler H, Stoeve J, Kluter H, Bieback K (2006). Comparative analysis of mesenchymal stem cells from bone marrow, umbilical cord blood, or adipose tissue. Stem Cells.

[31] Phinney DG, Kopen G, Righter W, Webster S, Tremain N, Prockop DJ (1999). Donor variation in the growth properties and osteogenic potential of human marrow stromal cells. Journal of Cellular Biochemistry.

[32] Zhou S, Greenberger JS, Epperly MW, Goff JP, Adler C, Leboff MS, Glowacki J (2008). Age-related intrinsic changes in human bone-marrow-derived mesenchymal stem cells and their differentiation to osteoblasts. Aging Cell.

[33] de Almeida DC, Ferreira MR, Franzen J, Weidner CI, Frobel J, Zenke M, Costa IG, Wagner W (2016). Epigenetic classification of human mesenchymal stromal cells. Stem Cell Reports.

[34] Praveen Kumar L, Kandoi S, Misra R, Vijayalakshmi S, Rajagopal K, Verma RS (2019). The mesenchymal stem cell secretome: A new paradigm towards cell-free therapeutic mode in regenerative medicine. Cytokine and Growth Factor Reviews.

[35] Madrigal M, Rao KS, Riordan NH (2014). A review of therapeutic effects of mesenchymal stem cell secretions and induction of secretory modification by different culture methods. Journal of Translational Medicine.

[36] Timmers L, Lim SK, Hoefer IE, Arslan F, Lai RC, van Oorschot AA, Goumans MJ, Strijder C, Sze SK, Choo A, Piek JJ, Doevendans PA, Pasterkamp G, de Kleijn DP (2011). Human mesenchymal stem cell-conditioned medium improves cardiac function following myocardial infarction. Stem Cell Research.

[37] Ahangar P, Mills SJ, Cowin AJ (2020). Mesenchymal stem cell secretome as an emerging cell-free alternative for improving wound repair. International Journal of Molecular Sciences.

[38] Duijvestein M, Vos AC, Roelofs H, Wildenberg ME, Wendrich BB, Verspaget HW, Kooy-Winkelaar EM, Koning F, Zwaginga JJ, Fidder HH, Verhaar AP, Fibbe WE, van den Brink GR, Hommes DW (2010). Autologous bone marrow-derived mesenchymal stromal cell treatment for refractory luminal Crohn's disease: Results of a phase I study. Gut.

[39] Le Blanc K, Frassoni F, Ball L, Locatelli F, Roelofs H, Lewis I, Lanino E, Sundberg B, Bernardo ME, Remberger M, Dini G, Egeler RM, Bacigalupo A, Fibbe W, Ringden O, Marrow T, B Developmental Committee of the European Group for (2008). Mesenchymal stem cells for treatment of steroid-resistant, severe, acute graft-versus-host disease: A phase II study. Lancet.

[40] Rad F, Ghorbani M, Mohammadi Roushandeh A, Habibi M, Roudkenar.  (2019). Mesenchymal stem cell-based therapy for autoimmune diseases: Emerging roles of extracellular vesicles. Molecular Biology Reports.

[41] Wang LT, Ting CH, Yen ML, Liu KJ, Sytwu HK, Wu KK, Yen BL (2016). Human mesenchymal stem cells (MSCs) for treatment towards immune- and inflammation-mediated diseases: Review of current clinical trials. Journal of Biomedical Science.

[42] Waterman RS, Tomchuck SL, Henkle SL, Betancourt AM (2010). A New Mesenchymal Stem Cell (MSC) paradigm: Polarization into a pro-inflammatory MSC1 or an immunosuppressive MSC2 phenotype. PLoS ONE.

[43] Mantovani A, Biswas SK, Galdiero MR, Sica A, Locati M (2013). Macrophage plasticity and polarization in tissue repair and remodelling. The Journal of Pathology.

[44] Li W, Ren G, Huang Y, Su J, Han Y, Li J, Chen X, Cao K, Chen Q, Shou P, Zhang L, Yuan ZR, Roberts AI, Shi S, Le AD, Shi Y (2012). Mesenchymal stem cells: A double-edged sword in regulating immune responses. Cell Death and Differentiation.

[45] Sato K, Ozaki K, Oh I, Meguro A, Hatanaka K, Nagai T, Muroi K, Ozawa K (2007). Nitric oxide plays a critical role in suppression of T-cell proliferation by mesenchymal stem cells. Blood.

[46] Liu X, Ren S, Ge C, Cheng K, Zenke M, Keating A, Zhao RC (2015). Sca-1+Lin-CD117- mesenchymal stem/stromal cells induce the generation of novel IRF8-controlled regulatory dendritic cells through Notch-RBP-J signaling. The Journal of Immunology.

[47] Carreras-Planella L, Monguio-Tortajada M, Borras FE, Franquesa M (2019). Immunomodulatory effect of MSC on B cells is independent of secreted extracellular vesicles. Frontiers in Immunology.

[48] Salem HK, Thiemermann C (2010). Mesenchymal stromal cells: Current understanding and clinical status. Stem Cells.

[49] Spaggiari GM, Capobianco A, Abdelrazik H, Becchetti F, Mingari MC, Moretta L (2008). Mesenchymal stem cells inhibit natural killer-cell proliferation, cytotoxicity, and cytokine production: Role of indoleamine 2,3-dioxygenase and prostaglandin E2. Blood.

[50] Spaggiari GM, Capobianco A, Becchetti S, Mingari MC, Moretta L (2006). Mesenchymal stem cell-natural killer cell interactions: Evidence that activated NK cells are capable of killing MSCs, whereas MSCs can inhibit IL-2-induced NK-cell proliferation. Blood.

[51] Bernardo ME, Fibbe WE (2013). Mesenchymal stromal cells: Sensors and switchers of inflammation. Cell Stem Cell.

[52] Le Blanc K, Mougiakakos D (2012). Multipotent mesenchymal stromal cells and the innate immune system. Nature Reviews Immunology.

[53] Toma C, Wagner WR, Bowry S, Schwartz A, Villanueva F (2009). Fate of culture-expanded mesenchymal stem cells in the microvasculature: In vivo observations of cell kinetics. Circulation Research.

[54] Gnecchi M, He H, Liang OD, Melo LG, Morello F, Mu H, Noiseux N, Zhang L, Pratt RE, Ingwall JS, Dzau VJ (2005). Paracrine action accounts for marked protection of ischemic heart by Akt-modified mesenchymal stem cells. Nature Medicine.

[55] Liang X, Ding Y, Zhang Y, Tse HF, Lian Q (2014). Paracrine mechanisms of mesenchymal stem cell-based therapy: Current status and perspectives. Cell Transplantation.

[56] Cuthbert RJ, Jones E, Sanjurjo-Rodriguez C, Lotfy A, Ganguly P, Churchman SM, Castana P, Tan HB, McGonagle D, Papadimitriou E, Giannoudis PV (2020). Regulation of angiogenesis discriminates tissue resident MSCs from effective and defective osteogenic environments. Journal of Clinical Medicine.

[57] Maacha S, Sidahmed H, Jacob S, Gentilcore G, Calzone R, Grivel JC, Cugno C (2020). Paracrine mechanisms of mesenchymal stromal cells in angiogenesis. Stem Cells International.

[58] Yao Z, Liu H, Yang M, Bai Y, Zhang B, Wang C, Yan Z, Niu G, Zou Y, Li Y (2020). Bone marrow mesenchymal stem cell-derived endothelial cells increase capillary density and accelerate angiogenesis in mouse hindlimb ischemia model. Stem Cell Research and Therapy.

[59] Bao L, Meng Q, Li Y, Deng S, Yu Z, Liu Z, Zhang L, Fan H (2017). C-Kit Positive cardiac stem cells and bone marrow-derived mesenchymal stem cells synergistically enhance angiogenesis and improve cardiac function after myocardial infarction in a paracrine manner. Journal of Cardiac Failure.

[60] Kinnaird T, Stabile E, Burnett MS, Shou M, Lee CW, Barr S, Fuchs S, Epstein SE (2004). Local delivery of marrow-derived stromal cells augments collateral perfusion through paracrine mechanisms. Circulation.

[61] Kossl J, Bohacova P, Hermankova B, Javorkova E, Zajicova A, Holan V (2021). Anti-apoptotic properties of mesenchymal stem cells in a mouse model of corneal inflammation. Stem Cells and Development.

[62] Islam MN, Das SR, Emin MT, Wei M, Sun L, Westphalen K, Rowlands DJ, Quadri SK, Bhattacharya S, Bhattacharya J (2012). Mitochondrial transfer from bone-marrow-derived stromal cells to pulmonary alveoli protects against acute lung injury. Nature Medicine.

[63] Ni S, Wang D, Qiu X, Pang L, Song Z, Guo K (2015). Bone marrow mesenchymal stem cells protect against bleomycin-induced pulmonary fibrosis in rat by activating Nrf2 signaling. International Journal of Clinical and Experimental Pathology.

[64] Stavely R, Nurgali K (2020). The emerging antioxidant paradigm of mesenchymal stem cell therapy. Stem Cells Translational Medicine.

[65] Xie C, Jin J, Lv X, Tao J, Wang R, Miao D (2015). Anti-aging effect of transplanted amniotic membrane mesenchymal stem cells in a premature aging model of Bmi-1 deficiency. Science and Reports.

[66] Chen MF, Lin CT, Chen WC, Yang CT, Chen CC, Liao SK, Liu JM, Lu CH, Lee KD (2006). The sensitivity of human mesenchymal stem cells to ionizing radiation. International Journal of Radiation Oncology Biology Physics.

[67] Kusuma GD, Carthew J, Lim R, Frith JE (2017). Effect of the microenvironment on mesenchymal stem cell paracrine signaling: Opportunities to engineer the therapeutic effect. Stem Cells and Development.

[68] Phelps J, Sanati-Nezhad A, Ungrin M, Duncan NA, Sen A (2018). Bioprocessing of mesenchymal stem cells and their derivatives: Toward cell-free therapeutics. Stem Cells International.

[69] Teixeira FG, Salgado AJ (2020). Mesenchymal stem cells secretome: Current trends and future challenges. Neural Regeneration Research.

[70] Sutton MGS, Sharpe N (2000). Left ventricular remodeling after myocardial infarction—Pathophysiology and therapy. Circulation.

[71] Stern CS, Lebowitz J (2010). Latest drug developments in the field of cardiovascular disease. International Journal of Angiology.

[72] Takada T, Hattori H, Kikuchi N, Ichihara Y, Saito S, Endo N, Iguchi S, Yoshida A, Kikuchi K, Niinami H, Hagiwara N, Nunoda S (2021). Heart transplant candidate with medical complexity in the era of prolonged left ventricular assist device support—a case report. Journal of Cardiology Cases.

[73] Mathiasen AB, Qayyum AA, Jorgensen E, Helqvist S, Fischer-Nielsen A, Kofoed KF, Haack-Sorensen M, Ekblond A, Kastrup J (2015). Bone marrow-derived mesenchymal stromal cell treatment in patients with severe ischaemic heart failure: A randomized placebo-controlled trial (MSC-HF trial). European Heart Journal.

[74] Mohamed SA, Howard L, McInerney V, Hayat A, Krawczyk J, Naughton S, Finnerty A, Holohan M, Duffy A, Moloney T, Kavanagh E, Burke P, Liew A, Tubassam M, Walsh SR, O'Brien T (2020). Autologous bone marrow mesenchymal stromal cell therapy for "no-option" critical limb ischemia is limited by karyotype abnormalities. Cytotherapy.

[75] Bolli R, Hare JM, Henry TD, Lenneman CG, March KL, Miller K, Pepine CJ, Perin EC, Traverse JH, Willerson JT, Yang PC, Gee AP, Lima JA, Moye L, Vojvodic RW, Sayre SL, Bettencourt J, Cohen M, Ebert RF, Simari RD, N Cardiovascular Cell Therapy Research (2018). Rationale and Design of the SENECA (StEm cell iNjECtion in cAncer survivors) Trial. American Heart Journal.

[76] Bolli R, Perin EC, Willerson JT, Yang PC, Traverse JH, Henry TD, Pepine CJ, Mitrani RD, Hare JM, Murphy MP, March KL, Ikram S, Lee DP, O'Brien C, Durand JB, Miller K, Lima JA, Ostovaneh MR, Ambale-Venkatesh B, Gee AP, Richman S, Taylor DA, Sayre SL, Bettencourt J, Vojvodic RW, Cohen ML, Simpson LM, Lai D, Aguilar D, Loghin C, Moye L, Ebert RF, Davis BR, Simari RD, N Cardiovascular Cell Therapy Research (2020). Allogeneic mesenchymal cell therapy in anthracycline-induced cardiomyopathy heart failure patients: The CCTRN SENECA Trial. JACC CardioOncol.

[77] Bolli R, Hare JM, March KL, Pepine CJ, Willerson JT, Perin EC, Yang PC, Henry TD, Traverse JH, Mitrani RD, Khan A, Hernandez-Schulman I, Taylor DA, DiFede DL, Lima JAC, Chugh A, Loughran J, Vojvodic RW, Sayre SL, Bettencourt J, Cohen M, Moye L, Ebert RF, Simari RD (2018). Rationale and design of the CONCERT-HF Trial (Combination of Mesenchymal and c-kit(+) cardiac stem cells as regenerative therapy for heart failure). Circulation Research.

[78] He X, Wang Q, Zhao Y, Zhang H, Wang B, Pan J, Li J, Yu H, Wang L, Dai J, Wang D (2020). Effect of intramyocardial grafting collagen scaffold with mesenchymal stromal cells in patients with chronic ischemic heart disease: A randomized clinical trial. JAMA Network Open.

[79] Gyurkocza B, Rezvani A, Storb RF (2010). Allogeneic hematopoietic cell transplantation: The state of the art. Expert Review of Hematology.

[80] Welniak LA, Blazar BR, Murphy WJ (2007). Immunobiology of allogeneic hematopoietic stem cell transplantation. Annual Review of Immunology.

[81] Mateos MK, O'Brien TA, Oswald C, Gabriel M, Ziegler DS, Cohn RJ, Russell SJ, Barbaric D, Marshall GM, Trahair TN (2013). Transplant-related mortality following allogeneic hematopoeitic stem cell transplantation for pediatric acute lymphoblastic leukemia: 25-year retrospective review. Pediatric Blood and Cancer.

[82] Assi MA, Pulido JS, Peters SG, McCannel CA, Razonable RR (2007). Graft-vs.-host disease in lung and other solid organ transplant recipients. Clinical Transplantation.

[83] Gulbahce HE, Brown CA, Wick M, Segall M, Jessurun J (2003). Graft-vs-host disease after solid organ transplant. American Journal of Clinical Pathology.

[84] Bacigalupo A, Milone G, Cupri A, Severino A, Fagioli F, Berger M, Santarone S, Chiusolo P, Sica S, Mammoliti S, Sorasio R, Massi D, Van Lint MT, Raiola AM, Gualandi F, Selleri C, Sormani MP, Signori A, Risitano A, Bonifazi F, O Gruppo Italiano Trapianto di Midollo (2017). Steroid treatment of acute graft-versus-host disease grade I: a randomized trial. Haematologica.

[85] Macmillan ML, Couriel D, Weisdorf DJ, Schwab G, Havrilla N, Fleming TR, Huang S, Roskos L, Slavin S, Shadduck RK, Dipersio J, Territo M, Pavletic S, Linker C, Heslop HE, Deeg HJ, Blazar BR (2007). A phase 2/3 multicenter randomized clinical trial of ABX-CBL versus ATG as secondary therapy for steroid-resistant acute graft-versus-host disease. Blood.

[86] Bloor AJC, Patel A, Griffin JE, Gilleece MH, Radia R, Yeung DT, Drier D, Larson LS, Uenishi GI, Hei D, Kelly K, Slukvin I, Rasko JEJ (2020). Production, safety and efficacy of iPSC-derived mesenchymal stromal cells in acute steroid-resistant graft versus host disease: A phase I, multicenter, open-label, dose-escalation study. Nature Medicine.

[87] Kurtzberg J, Abdel-Azim H, Carpenter P, Chaudhury S, Horn B, Mahadeo K, Nemecek E, Neudorf S, Prasad V, Prockop S, Quigg T, Satwani P, Cheng A, Burke E, Hayes J, Skerrett D, M-GS Group (2020). A Phase 3, Single-Arm, Prospective Study of Remestemcel-L, Ex Vivo culture-expanded adult human mesenchymal stromal cells for the treatment of pediatric patients who failed to respond to steroid treatment for acute graft-versus-host disease. Biology of Blood and Marrow Transplantation.

[88] Harris VK, Vyshkina T, Sadiq SA (2016). Clinical safety of intrathecal administration of mesenchymal stromal cell-derived neural progenitors in multiple sclerosis. Cytotherapy.

[89] Karussis D, Karageorgiou C, Vaknin-Dembinsky A, Gowda-Kurkalli B, Gomori JM, Kassis I, Bulte JW, Petrou P, Ben-Hur T, Abramsky O, Slavin S (2010). Safety and immunological effects of mesenchymal stem cell transplantation in patients with multiple sclerosis and amyotrophic lateral sclerosis. Archives of Neurology.

[90] Llufriu S, Sepulveda M, Blanco Y, Marin P, Moreno B, Berenguer J, Gabilondo I, Martinez-Heras E, Sola-Valls N, Arnaiz JA, Andreu EJ, Fernandez B, Bullich S, Sanchez-Dalmau B, Graus F, Villoslada P, Saiz A (2014). Randomized placebo-controlled phase II trial of autologous mesenchymal stem cells in multiple sclerosis. PLoS ONE.

[91] Petrou P, Kassis I, Levin N, Paul F, Backner Y, Benoliel T, Oertel FC, Scheel M, Hallimi M, Yaghmour N, Hur TB, Ginzberg A, Levy Y, Abramsky O, Karussis D (2020). Beneficial effects of autologous mesenchymal stem cell transplantation in active progressive multiple sclerosis. Brain.

[92] Uccelli A., Laroni A., Brundin L., Clanet M., Fernandez O., Nabavi S. M., Muraro P. A., Oliveri R. S., Radue E. W., Sellner J., Soelberg Sorensen P., Sormani M. P., Wuerfel J. T., Battaglia M. A., Freedman M. S. and Ms group. (2019). MEsenchymal StEm cells for Multiple Sclerosis (MESEMS): A randomized, double blind, cross-over phase I/II clinical trial with autologous mesenchymal stem cells for the therapy of multiple sclerosis. *Trials 20*, 263 10.1186/s13063-019-3346-z PMC6507027 31072380

[93] Hsu H., Siwiec, R. M. (2021). Knee Osteoarthritis. In: *StatPearls*. Treasure Island (FL).

[94] Lee WS, Kim HJ, Kim KI, Kim GB, Jin W (2019). Intra-articular injection of autologous adipose tissue-derived mesenchymal stem cells for the treatment of knee osteoarthritis: A Phase IIb, randomized, placebo-controlled clinical trial. Stem Cells Translational Medicine.

[95] Zhao X, Ruan J, Tang H, Li J, Shi Y, Li M, Li S, Xu C, Lu Q, Dai C (2019). Multi-compositional MRI evaluation of repair cartilage in knee osteoarthritis with treatment of allogeneic human adipose-derived mesenchymal progenitor cells. Stem Cell Research and Therapy.

[96] Powell SB, Silvestri JM (2019). Safety of intratracheal administration of human umbilical cord blood derived mesenchymal stromal cells in extremely low birth weight preterm infants. The Journal of Pediatrics.

[97] Lonardi R, Leone N, Gennai S, Trevisi Borsari G, Covic T, Silingardi R (2019). Autologous micro-fragmented adipose tissue for the treatment of diabetic foot minor amputations: A randomized controlled single-center clinical trial (MiFrAADiF). Stem Cell Research and Therapy.

[98] Moon KC, Suh HS, Kim KB, Han SK, Young KW, Lee JW, Kim MH (2019). Potential of allogeneic adipose-derived stem cell-hydrogel complex for treating diabetic foot ulcers. Diabetes.

[99] Park Y, Lee YJ, Koh JH, Lee J, Min HK, Kim MY, Kim KJ, Lee SJ, Rhie JW, Kim WU, Park SH, Moon SH, Kwok SK (2020). Clinical efficacy and safety of injection of stromal vascular fraction derived from autologous adipose tissues in systemic sclerosis patients with hand disability: A proof-of-concept trial. Journal of Clinical Medicine.

[100] Szczepanik E, Mierzewska H, Antczak-Marach D, Figiel-Dabrowska A, Terczynska I, Tryfon J, Krzesniak N, Noszczyk BH, Sawicka E, Domanska-Janik K, Sarnowska A (2020). Intrathecal infusion of autologous adipose-derived regenerative cells in autoimmune refractory epilepsy: Evaluation of safety and efficacy. Stem Cells International.

[101] Mattei A, Bertrand B, Jouve E, Blaise T, Philandrianos C, Grimaud F, Giraudo L, Aboudou H, Dumoulin C, Arnaud L, Revis J, Galant C, Velier M, Veran J, Dignat-George F, Dessi P, Sabatier F, Magalon J, Giovanni A (2020). Feasibility of first injection of autologous adipose tissue-derived stromal vascular fraction in human scarred vocal folds: A nonrandomized controlled trial. JAMA Otolaryngology. Head and Neck Surgery.

[102] Shi L, Huang H, Lu X, Yan X, Jiang X, Xu R, Wang S, Zhang C, Yuan X, Xu Z, Huang L, Fu JL, Li Y, Zhang Y, Yao WQ, Liu T, Song J, Sun L, Yang F, Zhang X, Zhang B, Shi M, Meng F, Song Y, Yu Y, Wen J, Li Q, Mao Q, Maeurer M, Zumla A, Yao C, Xie WF, Wang FS (2021). Effect of human umbilical cord-derived mesenchymal stem cells on lung damage in severe COVID-19 patients: A randomized, double-blind, placebo-controlled phase 2 trial. Signal Transduction and Targeted Therapy.

[103] Galipeau J (2013). The mesenchymal stromal cells dilemma–does a negative phase III trial of random donor mesenchymal stromal cells in steroid-resistant graft-versus-host disease represent a death knell or a bump in the road?. Cytotherapy.

